# Andrographolide Suppresses the Growth and Metastasis of Luminal-Like Breast Cancer by Inhibiting the NF-κB/miR-21-5p/PDCD4 Signaling Pathway

**DOI:** 10.3389/fcell.2021.643525

**Published:** 2021-06-23

**Authors:** Junchen Li, Lixun Huang, Zinan He, Minggui Chen, Yi Ding, Yuying Yao, Youfa Duan, Li Zixuan, Cuiling Qi, Lingyun Zheng, Jiangchao Li, Rongxin Zhang, Xiaoming Li, Jianwei Dai, Lijing Wang, Qian-Qian Zhang

**Affiliations:** ^1^School of Life Sciences and Biopharmaceutics, Guangdong Pharmaceutical University, Guangzhou, China; ^2^Guangdong Province Key Laboratory for Biotechnology Drug Candidates, Guangdong Pharmaceutical University, Guangzhou, China; ^3^Department of Pathology, People’s Hospital of Baoan District, Affiliated Baoan Hospital of Shenzhen, Southern Medical University, The Second Affiliated Hospital of Shenzhen University, Shenzhen, China; ^4^Guangzhou Medical University-Guangzhou Institute of Biomedicine and Health (GMU-GIBH) Joint School of Life Sciences, Guangzhou Medical University, Guangzhou, China; ^5^Key Laboratory for Major Obstetric Diseases of Guangdong Province, Key Laboratory of Reproduction and Genetics of Guangdong Higher Education Institutes, The Third Affiliated Hospital of Guangzhou Medical University, Guangzhou, China; ^6^The State Key Lab of Respiratory Disease, Guangzhou Institute of Respiratory Disease, The First Affiliated Hospital, Guangzhou Medical University, Guangzhou, China

**Keywords:** luminal-like breast cancer, andrographolide, growth, metastasis, NF-κB/miR-21-5p/PDCD4 signaling pathway

## Abstract

Tumor growth and metastasis are responsible for breast cancer-related mortality. Andrographolide (Andro) is a traditional anti-inflammatory drug used in the clinic that inhibits NF-κB activation. Recently, Andro has been found in the treatment of various cancers. Andro inhibits breast cell proliferation and invasion and induces apoptosis *via* activating various signaling pathways. Therefore, the underlying mechanisms with regard to the antitumor effects of Andro still need to be further confirmed. Herein, a MMTV-PyMT spontaneous luminal-like breast cancer lung metastatic transgenic tumor model was employed to estimate the antitumor effects of Andro on breast cancer *in vivo*. Andro significantly inhibited tumor growth and metastasis in MMTV-PyMT mice and suppressed the cell proliferation, migration, and invasion of MCF-7 breast cancer cells *in vitro*. Meanwhile, Andro significantly inhibited the expression of NF-κB, and the downregulated NF-κB reduced miR-21-5p expression. In addition, miR-21-5p dramatically inhibited the target gene expression of programmed cell death protein 4 (PDCD4). In the current study, we demonstrated the potential anticancer effects of Andro on luminal-like breast cancer and indicated that Andro inhibits the expression of miR-21-5p and further promotes PDCD4 *via* NF-κB suppression. Therefore, Andro could be an antitumor agent for the treatment of luminal-like breast cancer in the clinic.

## Introduction

Breast cancer is the most common malignant tumor among women with a rising incidence rate. It is one of the important causes of female deaths worldwide (Siegel et al., 2020). According to the gene expression profile, breast cancer can be divided into five subtypes: luminal-like (A and B subtypes), HER-2^+^, normal breast-like, and basal-like carcinomas (Sorlie et al., 2001, 2003). Luminal-like breast cancer accounts for more than 75% of the total incidence of breast cancer, and early luminal-like breast cancer is very sensitive to hormone endocrine therapy (Rosati et al., 2020). Luminal A and B breast cancers accounted for 37.1 and 8.6%, respectively, of patients with distant metastasis (Ihemelandu et al., 2008). Therefore, lumi-like breast cancer has a relatively high mortality rate due to distal metastasis. Monoclonal antibodies, tyrosine kinase inhibitors, and immunotherapy are commonly used to treat breast cancer in the clinic (Goldhirsch et al., 2011). However, there is no effective target therapy to treat metastatic luminal-like breast cancer. A recent study indicated that nuclear factor-kappa B (NF-κB) can be considered a valid drug target in cancers (Labbozzetta et al., 2020). The expression of NF-κB is highly correlated with the occurrence, late development, and metastasis of breast cancer (Yamamoto et al., 2013). Inhibition of NF-κB effectively prevents the growth and invasion of tumor cells (Liu et al., 2019). Therefore, whether NF-κB could be an effective therapeutic target in metastatic luminal-like breast cancer still needs to be further investigated.

Multiple cancer-related signaling pathways are involved in tumorigenesis and considered as new targets for breast cancer therapy (Li et al., 2017; Tewari et al., 2019, 2021). In recent years, traditional Chinese medicine has attracted increasing attention for anticancer therapy due to its potential to modulate multiple cancer-related signaling pathways (Yan et al., 2017). Andrographolide (Andro) is a diterpene lactone compound extracted from *Andrographis paniculata* and acts as an NF-κB inhibitor (Xia et al., 2004). *A. paniculata* is a classic Chinese herb with anti-inflammatory properties. It was demonstrated that Andro possesses anticancer activity by inducing tumor cell apoptosis, inhibiting tumor angiogenesis, affecting tumor cell cycle, and regulating autoimmune mechanisms (Zhang et al., 2014a, b). Recently *in vitro* studies indicated that Andro inhibits 12-*O*-tetradecanoylphorbol-13-acetate (TPA)-induced MCF-7 luminal-like breast cancer cell migration and invasion (Chao et al., 2013). Andro inactivates matrix metalloproteinase-9 (MMP9) *via* inhibition of the extracellular signal-regulated kinase (ERK) 1/2 and PI3K/Akt signaling that further suppresses the DNA binding activity of activator protein-1 (AP-1) and NF-κB (Chao et al., 2013). Meanwhile, Andro inhibits MDA-MB-231 triple-negative breast cancer cell proliferation and metastasis by suppressing MMP9 and induces apoptosis through arresting cell cycle progression by inducing changes in the Bax/Bcl-2 ratio (Zhai et al., 2015; Banerjee et al., 2016). However, *in vivo* research on the anti-breast cancer effects of Andro and the underlying mechanisms still needs to be further clarified.

MicroRNAs (miRNAs) have emerged as central posttranscriptional regulators of gene expression and further regulate tumor progression. It has been reported that miR-21-5p overexpression promotes tumor growth and metastasis in breast cancer (Zhang et al., 2014c; Petrovic, 2016). The tumor suppressor programmed cell death 4 (PDCD4) is downregulated in breast cancer and acts as a functional target of miR-21-5p in breast cancer cells (Chen et al., 2015; Tao et al., 2019). Furthermore, miR-21-5p/PDCD4 signaling has been reported to be involved in paclitaxel-resistant breast cancer cells (Tao et al., 2019). NF-κB binds to the promoter region of miR-21-5p and activates the transcription of miR-21-5p, further enhancing the survival of MDA-MB-231 cells (Polytarchou et al., 2011). In our previous report, we demonstrated that Andro inhibits miR-21-5p/TIMP3 signaling to suppress angiogenesis (Dai et al., 2017). However, whether the interplay between miR-21-5p and PDCD4 is involved in the Andro-induced inhibition of breast cancer growth and metastasis of luminal-like breast cancer remains undefined.

Here, we demonstrated that NF-κB promotes miR-21-5p expression and then further inhibits PDCD4 expression in luminal-like breast cancer. NF-κB/miR-21-5p/PDCD4 signaling promotes the tumor growth and metastasis of luminal-like breast cancer. Andro inhibits the tumor growth and metastasis of luminal-like breast cancer *in vitro* and *in vivo* mainly by targeting NF-κB/miR-21-5p/PDCD4 signaling.

## Materials and Methods

### Reagents and Antibodies

Andro (365645) and ammonium pyrrolidinedithiocarbamate (APDC; NF-κB inhibitor, P8765) were obtained from Sigma-Aldrich (St. Louis, MO, United States) and were dissolved in dimethyl sulfoxide (DMSO). Cell Counting Kit-8 (CCK-8) was purchased from Beyotime (Shanghai, China). Rabbit anti-Ki67 (ab15580) was purchased from Abcam (Cambridge, MA, United States), rabbit anti-GAPDH (2118) was purchased from Cell Signaling Technology, Inc. (Danvers, MA, United States), rabbit anti-PDCD4 (BM5208) and rabbit anti-EGN (endoglin, BA2227) were both from Boster (Wuhan, China), and rabbit anti-NF-κB p65 (abs131170) and rabbit anti-phospho-NF-κB p65 (Ser536, abs130624) were purchased from Absin (Shanghai, China). All the miRNA mimics and inhibitors and the negative control (NC) RNAs were purchased from RiboBio Co., Ltd. (Guangzhou, China). All the primers were synthesized by Sangon Biotech Co., Ltd. (Shanghai, China).

### Patients and Tissue Samples

A total of 12 cases of tumor tissues of female patients with luminal-like breast cancer, with a median age of 51.8 years (ranging from 40 to 66 years), and their matched non-tumorous breast tissues, as well as the corresponding clinical data, were collected from the Department of Pathology, People’s Hospital of Baoan District. Pathologic diagnosis was performed by two independent pathologists based on the guidelines of the International Union Against Cancer (UICC). The study has been approved by the Institutional Ethics Committee of People’s Hospital of Baoan District of Shenzhen (BYL20200902).

### Animal Manipulations and Treatment

MMTV-PyMT mice (stock no. 002374) were purchased from the Jackson Laboratory (Bar Harbor, ME, United States) and housed in the Undergraduate Laboratory Animal Center of Guangdong Pharmaceutical University in an environmentally controlled condition with a 12:12-h light/dark cycle, temperature of 22 ** ±** 2°C, and humidity of 60 ** ±** 5%. All animal experiments were conducted according to the Guide for the Care and Use of Laboratory Animals by the National Academy of Sciences (NIH publication no. 80-23, revised 1996). The protocols were approved by the Undergraduate Laboratory Animal Center ethics committee of Guangdong Pharmaceutical University (gdpulac2020014). All possible efforts were made to minimize animal suffering.

MMTV-PyMT mice (9 weeks old, females) were randomly divided into two groups with intraperitoneal injection of Andro (5 μg/g) or DMSO twice a week for 4 weeks, in accordance with previous reports (Handa and Sharma, 1990; Zhang et al., 2014a, b; Peng et al., 2018). The length (*L*) and width (*W*) of tumors were measured with calipers, and the tumor volumes (*V*) were measured twice a week and calculated as follows: *V* = (*L* × *W*^2^) × 0.5236. Mice were sacrificed after treatment, the tumors were peeled off and weighted, and the lung tissues were removed and fixed in Bouin’s solution.

### Cells

MCF-7 cells were kindly provided by the Stem Cell Bank, Chinese Academy of Sciences (Shanghai, China), and maintained in a humidified incubator with 5% CO_2_ at 37°C. All cells were cultured in minimum essential medium (MEM; Invitrogen, Carlsbad, CA, United States) supplemented with 10% fetal bovine serum (FBS; Gibco, Waltham, MA, United States), 1% glutamax (Invitrogen), 1% non-essential amino acids (NECC; Invitrogen), 0.01 mg/ml human recombinant insulin (91077C, Sigma-Aldrich), 100 U/ml penicillin, and 100 μg/ml streptomycin (Gibco).

### Cell Viability Assay

MCF-7 cells (1.5 × 10^3^ cells/well) were seeded into 96-well plates and treated with the indicated concentrations of Andro and DMSO. Cell viability and proliferation were detected at the indicated time after treatment with Andro or DMSO using the CCK-8 reagent (Beyotime Institute Biotechnology, Shanghai, China). The CCK-8 reagent (10 μl) was added to each well and further incubated at 37°C for 3 h, and then the solution absorption was read at 450 nm by a spectrophotometer.

### Cell Proliferation Assay

The colony formation assay was used to assess the cell proliferation ability. MCF-7 cells (3,000 cells/well) were seeded into six-well plates and then Andro (70 μM) or DMSO was added into the wells 24 h later. The colonies were fixed in 4% paraformaldehyde, stained with 0.1% crystal violet solution after 7 days, and the number of colonies was counted. The data shown were representative of three independent experiments, in which each treatment was assayed in duplicate.

### Wound Healing Assay

MCF-7 cells were seeded into 12-well plates at a density of 5 × 10^5^ cells per well. After 12 h, a scratch was created with a pipette tip in the center of the well and washed with phosphate buffer saline (PBS) to remove the scraped cells. Then, the cells were treated with Andro (70 μM) or DMSO and photographed at 0 and 12 h posttreatment. The migrated distances of cells from the wound edge since 0 h were measured.

### Cell Migration and Invasion Assay

Transwell chambers (8 μM pores; Costar, Cambridge, MA, United States) uncoated or coated with 50 μl Matrigel (1:20 dilution with serum-free MEM; BD Biosciences, San Jose, CA, United States) were used to detect cell migration and invasion ability. The uncoated or coated chambers were placed in a 24-well plate at 37°C for 7 h. Then, the chambers were removed from the well of the 24-well plate with 600 μl MEM with 10% serum and the MCF-7 cells (2 × 10^5^ cells) mixed with Andro (70 μM) or DMSO were added into the upper chambers. The membranes of the chambers were fixed with methanol and stained with 1% crystal violet after 20 h incubation, and then the cells on the upper membranes of the chambers were wiped. The membranes were photographed and the number of cells that migrated and invaded through the membranes was counted.

### Real-Time Quantitative PCR Assay

Total RNAs from MCF-7 cells and the tumor tissue treated with Andro or DMSO were extracted using TRIzol reagent (Invitrogen). Briefly, total RNA (1,000 ng) was used for reverse transcription using a PrimeScript^TM^ RT Reagent Kit (TaKaRa, Shiga, Japan). Then, real-time quantitative PCR was performed using the SYBR^®^ Premix Ex Taq^TM^ II (Tli RNaseH Plus) PCR Kit (TaKaRa, Japan) on a LightCycler^®^ 96 PCR machine (Roche Diagnostics, Basel, Switzerland). All the experiments were carried out according to the manufacturers’ instructions. All samples were tested in triplicate and repeated three times each. All the primers that were used for reverse transcription and PCR are presented in Supplementary Table 1.

### Histological, Immunohistochemical, and Immunoblotting Analysis

The fixed lung tissues of Andro- or DMSO-treated MMTV-PyMT mice were embedded in paraffin, cut into 3-μM sections, and stained with hematoxylin and eosin (H&E) for histological analyses or incubated with ki67 antibodies for immunohistochemical (IHC) assay. The total proteins from the tumor tissues and MCF-7 cells were harvested by RIPA buffer and separated by 10% sodium dodecyl sulfate–polyacrylamide gel electrophoresis (SDS-PAGE). Then, the proteins were transferred onto polyvinylidene fluoride (PVDF) membranes and blocked with 10% non-fat milk in Tris-buffered saline containing 0.2% Tween 20 (TBST) for 1 h at room temperature. The blocked membranes were incubated with relevant primary antibodies and horseradish peroxidase (HRP)-conjugated secondary antibody. The bands were visualized with an enhanced chemiluminescence (ECL) reagent. GAPDH served as a loading control.

### Statistical Analysis

All data are represented as the mean ** ±** standard deviation (SD). Student’s two-sided *t*-test was used to analyze statistical differences between the two groups. The protein expression levels in the IHC slices were determined using IPP software (Media Cybernetics, Inc., Rockville, MD, United States) and those in the immunoblotting bands were analyzed using Quantity One software (Bio-Rad Laboratories, Inc., Hercules, CA, United States). The protein band intensities were quantified to those of GAPDH. *P* **<** 0.05 was considered the statistically significant difference between the two groups.

## Results

### NF-κB Is Highly Expressed in Luminal-Like Breast Cancer Tissues

**FIGURE 1:**
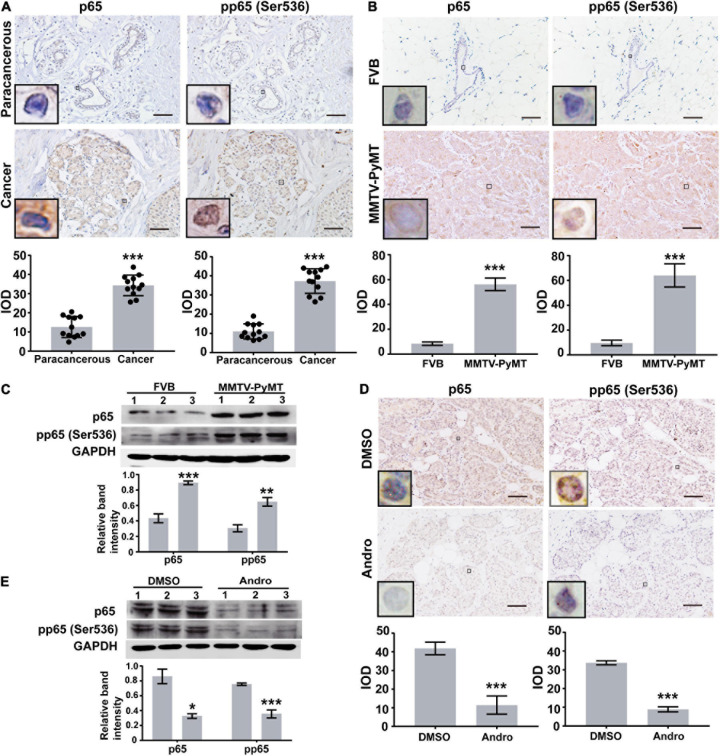
Expression levels of NF-κB. Immunohistochemistry (IHC) assay analysis the expression levels of p65 and pp65(Ser536) in **(A)** human luminal-like breast cancer tissues and the matched surrounding non-cancerous breast tissues (paracancerous, *n* = 12) and **(B)** tumor tissues of spontaneous luminal-like breast cancer mice (MMTV-PyMT) and breast tissues of matched background mice (FVB, *n* = 3). **(C)** Immunoblotting analysis in mice breast tissue lysates. **(D)** Expressions of p65 and pp65 (Ser536) in the tumor tissues of dimethyl sulfoxide (DMSO)- or andrographolide (Andro)-treated MMTV-PyMT mice (*n* = 3). **(E)** Immunoblotting analysis of p65 and pp65 (Ser536) expression in MCF-7 cells. Quantitative data are expressed as the mean ± SD. Significant effect: **P < 0.01; ***P < 0.001. *Scale bar*, 50 μM.

Previous reports indicated that Andro inhibits NF-κB activation in inflammation and cancers (Xia et al., 2004). Therefore, we first randomly selected 12 pairs of luminal-like breast cancer tissues and non-cancerous breast tissues (nine cases of luminal-like A type and three cases of luminal-like B type) to evaluate the expression of NF-κB in human luminal-like breast cancer by immunohistochemical staining. The expressions of p65 and phospho-p65 [pp65(Ser536)] in human luminal-like breast cancer were significantly higher than those of the matched non-cancerous breast tissues (Figure 1A). In addition, we found upregulated expressions of p65 and pp65(Ser536) in tumor tissues of MMTV-PyMT mice than in FVB mice (Figures 1B,C). Andro significantly inhibited the expressions of p65 and pp65 (Ser536) in the tumor tissues of MMTV-PyMT mice and MCF-7 cells (Figures 1D,E).

### Andro Inhibits Tumor Growth of Luminal-Like Breast Cancer in MMTV-PyMT Mice

**FIGURE 2:**
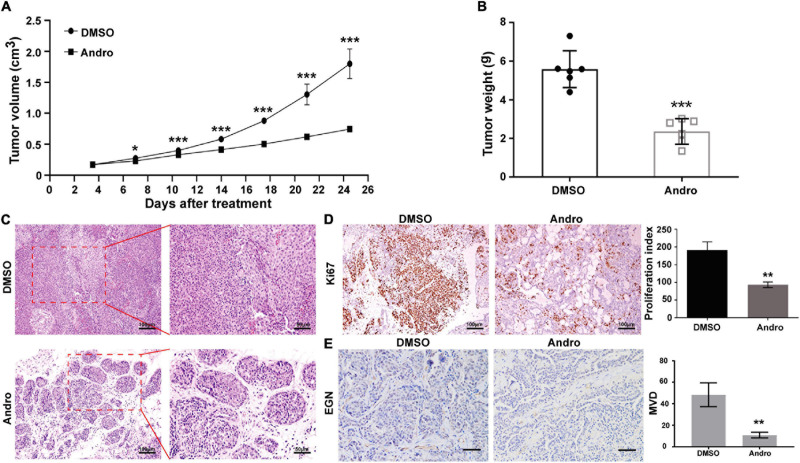
Andrographolide (Andro) suppresses the tumor growth of MMTV-PyMT mice. **(A)** Tumor volume, **(B)** tumor weight, **(C)** tumor morphology, **(D)** Ki67, and **(E)** EGN expression detected in treatment with Andro or dimethyl sulfoxide (DMSO). Quantitative data are expressed as the mean ± SD (*n* = 6). Significant effect: *P < 0.05; **P < 0.01; ***P < 0.001.

Female MMTV-PyMT mice were randomly divided into two groups and treated with Andro or DMSO twice a week for 4 weeks. The tumor volume was calculated twice a week. Andro significantly inhibited tumor growth after a 7-day treatment compared to DMSO (Figure 2A). After treatment, the tumor tissues were peeled off and weighed. The tumor weight of each mouse was markedly suppressed by Andro treatment compared to DMSO (Figure 2B). To explore the effect of Andro on luminal-like breast cancer development, a detailed histological analysis of the tumor tissues of DMSO- and Andro-treated MMTV-PyMT mice was performed. As shown in Figure 2C, Andro slowed down the pathological process of tumor tissues in MMTV-PyMT mice. Advanced-stage cancer invaded the tissues and extensive necrotic area were identified in the DMSO-treated mice. However, the structure of most of the tumor tissue basement membrane was complete in Andro-treated MMTV-PyMT mice. We further detected the cell proliferation index in the tumor tissues, and the results showed that cells expressing Ki67, a cell proliferation marker, were notably decreased in the Andro-treated group compared to the DMSO-treated group (Figure 2D). In our previous report, we demonstrated that Andro can inhibit angiogenesis, which can promote tumor growth and metastasis (Dai et al., 2017). EGN (CD105), a marker of angiogenic endothelial cells, is expressed specifically in tumor angiogenesis, including breast cancer (Minhajat et al., 2006). Therefore, we further examined the expression of EGN in tumor tissues and found that Andro can significantly inhibit the number of EGN-positive cells in tumor tissues. Furthermore, the vascular density in tumor tissues was significantly suppressed by Andro compared to treatment by DMSO (Figure 2E). Together, these results revealed that Andro inhibits the tumor growth of luminal-like breast cancer.

### Andro Suppresses Tumor Metastasis of Luminal-Like Breast Cancer *in vivo*

**FIGURE 3:**
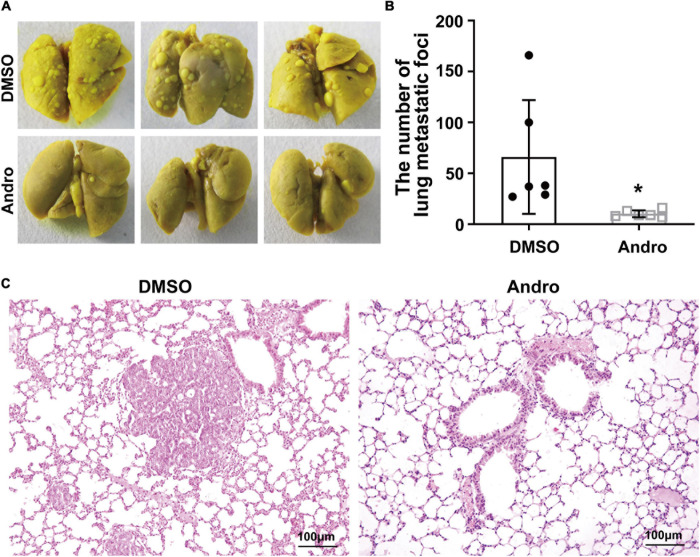
Andrographolide (Andro) inhibits tumor metastasis to the lung in MMTV-PyMT mice. **(A)** Gross observation of metastatic foci on the surface of the lungs. **(B)** Quantification of metastatic foci. **(C)** Histological images of hematoxylin and eosin (H&E)-stained lung tissue sections. Quantitative data are expressed as the mean ± SD (*n* = 6). Significant effect: *P < 0.05.

The MMTV-PyMT transgenic mouse model is a widely used spontaneous luminal-like breast cancer mouse model with metastasis to the lungs (Fluck and Schaffhausen, 2009). Therefore, we further examined the anti-metastatic effect of Andro in luminal-like breast cancer. After treatment with Andro or DMSO, the lung tissues of mice were removed and fixed with Bouin’s solution. Multiple large metastatic foci on the surface of the lung tissues were observed in DMSO-treated MMTV-PyMT mice, but not in the lung tissues of the Andro-treated mice (Figure 3A). Then, the numbers of metastatic foci on the surface of the lung tissues were counted; the results showed that Andro significantly inhibits tumor metastasis to the lungs compared to DMSO (Figure 3B). In addition, the lung tissues were embedded in paraffin and stained with H&E. The metastatic nodules on the tissue sections were larger in the DMSO-treated group than that in the Andro-treated group (Figure 3C). All of the data demonstrated that Andro inhibits the tumor metastasis of luminal-like breast cancer.

### Andro Inhibits Cell Proliferation of MCF-7 Cells *in vitro*

**FIGURE 4:**
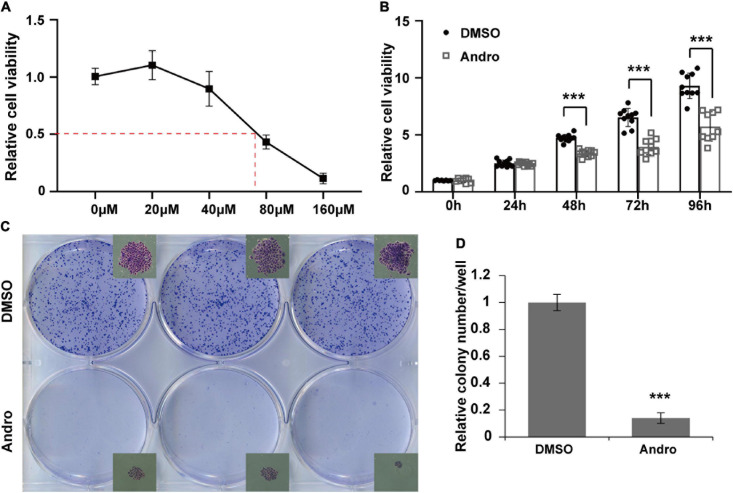
Andrographolide (Andro) inhibits viability and proliferation of MCF-7 cells. **(A)** Dose-dependent effect of andrographolide (Andro) on MCF-7 cell viability at 48 h (IC_50_ = 70 μM). **(B)** Treatment duration effect of Andro (70 μM) on MCF-7 viability. **(C)** Colony formation assay. **(D)** Colony counts. Quantitative data are expressed as the mean ± SD (*n* = 3). Significant effect: ***P < 0.001.

MCF-7 luminal-like breast cancer cells were further employed to evaluate the inhibition effect of Andro on tumor cells *in vitro*. MCF-7 cells were treated with different concentrations of Andro. Andro significantly suppressed MCF-7 cell viability in a dose-dependent manner, with an IC_50_ of 70 μM for 48 h treatment (Figure 4A). In addition, the IC_50_ concentration of Andro inhibited cell viability in a time-dependent manner (Figure 4B). Next, colony formation assay was used to detect the long-term effect of Andro on cell proliferation. MCF-7 cells were seeded in a low density and treated with Andro (70 μM) or DMSO. After 7 days of treatment, Andro-treated colonies were smaller than the DMSO-treated ones (Figure 4C). Andro significantly decreased the number of colonies compared to DMSO (Figure 4D). All the results indicated that Andro could significantly inhibit the cell proliferation of MCF-7 luminal-like breast cancer cells in a dose- and time-dependent manner.

### Andro Inhibits Cell Migration and Invasion of MCF-7 Cells *in vitro*

**FIGURE 5:**
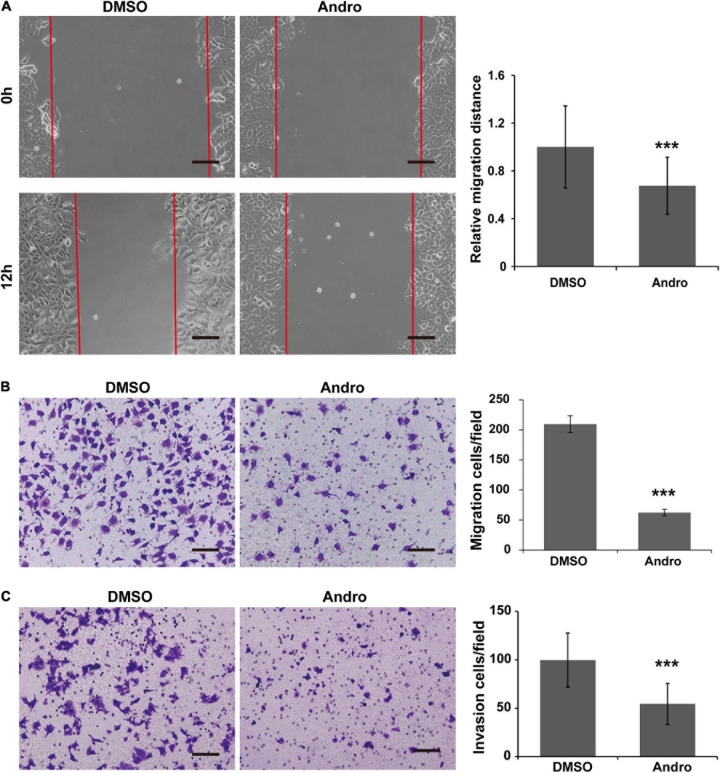
Andrographolide (Andro) inhibits migration and invasion of MCF-7 cells. **(A)** Wound healing assay for MCF-7 cell migration. Transwell assay for the migration **(B)** and invasion **(C)** potential of MCF-7 cells. Quantitative data are expressed as the mean ± SD (*n* = 3). Significant effect: ***P < 0.001. *Scale bar*, 100 μM.

As shown in Figure 5A, the cell migration distances were markedly decreased at 12 h posttreatment with Andro compared to DMSO. In addition, the MCF-7 cell migration and invasion abilities were inhibited by Andro. The migrated and invaded cells through the Matrigel-uncoated or Matrigel-coated membranes of the chambers were less in the Andro-treated group than those in the DMSO-treated group (Figures 5B,C). These results demonstrated that Andro suppresses the migration and invasion of MCF-7 luminal-like breast cancer cells.

### The NF-κB/miR-21-5p/PDCD4 Signaling Pathway Is Involved in Andro-Mediated Inhibition of MCF-7 Cell Proliferation, Migration, and Invasion

**FIGURE 6:**
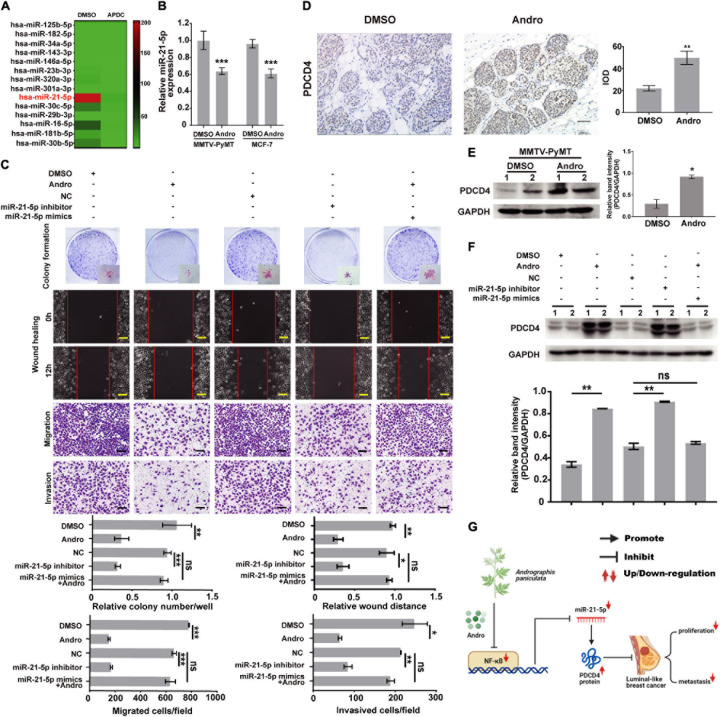
Andrographolide (Andro) inhibits breast cancer cell proliferation, migration, and invasion *via* NF-κB/miR-21-5p/PDCD4 signaling. **(A)** Heat map of the qRT-PCR data of miRNA expression in MCF-7 cells with or without NF-κB inhibition. APDC: NF-κB inhibitor. **(B)** miR-21-5p expression in breast cancer tissue and MCF cells was also detected using qRT-PCR. miR-21-5p was markedly suppressed by Andro in MCF-7 cells and MMTV-PyMT mice. **(C)** Colony formation, wound healing, Transwell migration, and Matrigel invasion assay. Immunohistochemistry **(D)** and Immunoblotting assay **(E)** for PDCD4 expression in DMSO- and Andro-treated MMTV-PyMT mice. **(F)** Immunoblotting analysis for PDCD4 expression in MCF-7 cells. **(G)** Schematic of the mechanisms of Andro-mediated effect on luminal-like breast cancer growth and metastasis. Quantitative data are expressed as the mean ± SD, *n* = 3. Significant effect: *P < 0.05, **P < 0.01, ***P < 0.005. *ns*, no significant effect. *Scale bar*, 100 μM.

It has been reported that NF-κB regulates multiple miRNA expressions in various types of cancer cells (Shin et al., 2011; Wei et al., 2014). As shown in Figure 6A, miR-21-5p was significantly inhibited by the inactivation of NF-κB in MCF-7 cells. Previous studies have reported that NF-κB binding sites in the promoter of miR-21-5p transcription regulated the expression of miR-21-5p (Polytarchou et al., 2011; Yang et al., 2017). We demonstrated that Andro inhibits the expression of NF-κB in luminal-like breast cancer; however, whether Andro can inhibit miR-21-5p expression in luminal-like breast cancer still needs to be confirmed. In addition, we also found that the expression of miR-21-5p also decreased in MCF-7 cells and MMTV-PyMT mice treated with Andro compared with DMSO (Figure 6B). As shown in Figure 6C, inhibition of miR-21-5p significantly inhibited MCF-7 cell proliferation, migration, and invasion. Meanwhile, the overexpression of miR-21-5p in MCF-7 cells abolished the Andro-mediated inhibition of cell proliferation, migration, and invasion.

We further explored the target gene of miR-21-5p in breast cancer cells. In a previous study, we has confirmed that miR-21-5p directly binds to the 3′-UTR region of PDCD4 through dual-luciferase reporter assays (Yao et al., 2009). In this study, we demonstrated that Andro significantly upregulated the expression of PDCD4 in MMTV-PyMT mice and MCF-7 cells (Figures 6D–F). Andro treatment or miR-21-5p knockdown markedly upregulated PDCD4 expression compared to the control groups. Andro-induced PDCD4 expression in MCF-7 was abolished by miR-21-5p mimic treatment (Figure 6F). These results indicate that Andro inactivates NF-κB signaling to inhibit the expression of miR-21-5p, and the inhibition of miR-21-5p stimulates PDCD4 expression to inhibit luminal-like breast cancer growth, metastasis, and invasion.

## Discussion

Cost-effective drugs for luminal-like breast cancer treatment are still in high demand. In this study, we have demonstrated the promising therapeutic potential of Andro for luminal-like breast cancer and explored the underlying mechanisms (Figure 6G). We verify that NF-κB was expressed at a higher level in tumor tissues compared to that of matched non-cancerous tissues of luminal-like breast cancer. *In vivo* and *in vitro* studies demonstrated that Andro, a potent inhibitor of NF-κB, inhibits the tumor growth and metastasis of luminal-like breast cancer. Previous reports and our work further demonstrated that NF-κB inhibits PDCD4 expression in breast cancer cells *via* regulating miR-21-5p expression. These results indicate NF-κB/miR-21-5p/PDCD4 signaling was involve in the inhibition of tumor growth and metastasis by Andro in luminal-like breast cancer. Andro, a potent NF-κB inhibitor, which could be a possible drug to treat luminal-like breast cancer in the clinic.

Endocrine therapy is recommended as the first-line treatment in the clinic for luminal-like breast cancer, which is a type of hormone receptor-positive breast cancer (Cortes et al., 2020). More than half of luminal-like breast cancer patients exhibit treatment-related toxicity during chemotherapy, and some patients develop drug tolerance during endocrine therapy (Ignatiadis and Sotiriou, 2013). Both chemotherapy and endocrine therapy are not effective for metastasized luminal breast cancer (Kimbung et al., 2016). Therefore, this study aimed to explore a possible new molecular target for luminal-like breast cancer. A previous report has indicated NF-κB as a possible therapeutic target for various cancers (Labbozzetta et al., 2020). The tumor tissues of patients with luminal-like breast cancers and MMTV-PyMT mice showed NF-κB overexpression. These findings indicate NF-κB as a therapeutic target for luminal-like breast cancer.

A targeted therapy strategy based on molecular characteristics is well used for the treatment of hormone receptor- or HER2-positive breast cancer (Ithimakin et al., 2013). Luminal-like breast cancers, including the luminal A, luminal B HER2-negative, and luminal B HER2-positive subtypes, are often resistant to anti-hormone therapy (Santiago-Ortiz and Schaffer, 2016). Although anti-HER2 therapies have been shown to improve the outcomes of patients, these can only be used to treat the luminal B HER2-positive subtype in luminal-like breast cancers (Tagliabue et al., 2010). *A. paniculata* Nees belongs to the family Acanthaceae and is an important traditional herbal medicine that is widely used in many Asian countries such as China, India, and others in Southeast Asia (Sun et al., 2019). Andrographolide (Andro), a diterpene lactone, is the major bioactive component extracted from *A. paniculata* Nees, which has been traditionally used for thousands of years to treat bacterial infections and inflammatory diseases (Zhang et al., 2020). In recent years, it has been demonstrated that Andro also possesses anticancer, antidiabetic, antimalarial, anti-HIV, and anti-angiogenic pharmacological effects (Sukardiman et al., 2007; Di Leva et al., 2014; Kang et al., 2018). Previous reports indicated that Andro inhibits cell growth, migration, and invasion and induces apoptosis in multiple types of breast cancer cells, including luminal-type (MCF-7 and TD-47) and triple-negative (MDA-MB-231 and 4T1) breast cancer cells, through various signaling pathways (Sukardiman et al., 2007; Chao et al., 2013; Zhai et al., 2015; Kang et al., 2018). It has been reported that Andro acts as an NF-κB inhibitor to suppress various cancers (Zhang et al., 2014a, b). Andro-mediated inhibition of NF-κB signaling has only been reported in MDA-MB-231 cells (Zhai et al., 2015). In this study, we further demonstrated that Andro inhibits the tumor growth and metastasis of luminal-like breast cancer by suppressing cell viability and cell migration and invasion *in vivo* and *in vitro*. Otherwise, our previous study demonstrated that Andro inhibits angiogenesis, which is essential for tumor growth and metastasis (Dai et al., 2017). We further confirmed that tumor angiogenesis is inhibit by Andro in the tumor tissues of MMTV-PyMT mice, which is consistent with our previous report indicated above. All of the data showed that Andro inhibits cancer development in luminal-like breast cancer, which may be through not only inhibiting tumor cell proliferation and invasion but also by suppressing angiogenesis.

Aberrant expressions of miRNAs are associated with breast cancer pathogenesis. Recent studies have indicated that miR-21-5p is an important oncogenic miRNA. miR-21-5p is consistently overexpressed in many solid tumors, including breast cancer (Zhang et al., 2014c). It has been shown that miR-21-5p specifically targets and inhibits tumor suppressor expression to promote cell proliferation, migration, and invasion in breast cancer (Wang et al., 2019). PDCD4 is a significant functional target of miR-21-5p in various types of cancers (Chen et al., 2015; Tao et al., 2019). *In vitro* studies indicated that miR-21-5p/PDCD4 signaling promotes cell proliferation in MCF-7 luminal-like breast cancer cells and induces the invasion and metastasis of MDA-MB-231 triple-negative breast cancer cells (Frankel et al., 2008; Zhu et al., 2008). Multiple miRNAs, including miR-21-5p, are validated as transcriptional targets of NF-κB (Polytarchou et al., 2011; Shin et al., 2011; Wei et al., 2014). In this study, NF-κB-inactivated luminal-like breast cancer cells demonstrated significant downregulation of miR-21-5p. In addition, NF-κB/miR-21-5p/PDCD4 signaling was involved in tumor growth and metastasis.

This study detected the crosstalk between NF-κB and miR-21-5p. Andro showed an inhibitory effect on luminal-like breast cancer growth and metastasis *via* the downregulation of NF-κB and miRNA-21-5p. Reports from the literature indicated that breast cancer development may be dependent on the crosstalk between NF-κB and other signaling pathways, including signal transducer and activator of transcription 3 (STAT3), glycogen synthase kinase 3 beta (GSK3-β), tumor suppressor p53, miRNAs, lncRNA, and the Wnt/β-catenin pathway (Moreau et al., 2011; Schneider and Kramer, 2011; Keklikoglou et al., 2012; Yu et al., 2014; Liu et al., 2015; Tewari et al., 2021). This is the first study to demonstrate the therapeutic effect of Andro on luminal-like breast cancer *via* targeting PDCD4 through the downregulation of NF-κB/miR-21-5p signaling. However, the interactions between NF-κB and other signaling pathways and that of miR-21-5p and its other targets involved in Andro-mediated tumor inhibition of luminal-like breast cancer need to be further explored.

## Data Availability Statement

The original contributions presented in the study are included in the article/Supplementary Material, further inquiries can be directed to the corresponding author/s.

## Ethics Statement

The studies involving human participants were reviewed and approved by the Institutional Ethics Committee of People’s Hospital of Baoan District of Shenzhen. The patients/participants provided their written informed consent to participate in this study. The animal study was reviewed and approved by the Undergraduate Laboratory Animal Center Ethics Committee of Guangdong Pharmaceutical University.

## Author Contributions

Q-QZ, JD, and LW conceptualized and supervised the study. CQ, LZ, JiL, and JD helped with the methodology. JuL, YY, ZH, MC, YDi, and ZL helped with the software. Q-QZ and JuL did the validation, wrote the manuscript—revised draft preparation, and contributed to the visualization. JuL, ZH, MC, YY, YDu, XL, and ZL did the formal analysis. JuL, LH, ZH, MC, YDi, YY, YDu, and ZL did the investigation. Q-QZ contributed to resources. Q-QZ, JuL, LH, YDu, and YY curated the data. Q-QZ, LW, JD, and RZ administered the project and helped with funding acquisition. All authors have read and agreed to the published version of the manuscript.

## Conflict of Interest

The authors declare that the research was conducted in the absence of any commercial or financial relationships that could be construed as a potential conflict of interest.
