# Supplementary material for: Andrographolide Suppresses the Growth and Metastasis of Luminal-Like Breast Cancer by Inhibiting the NF-κB/miR-21-5p/PDCD4 Signaling Pathway
Source: Front Cell Dev Biol. 2021 Jun 23;9:643525. doi: 10.3389/fcell.2021.643525 (PMC8261247; doi:10.3389/fcell.2021.643525)
Supplement: Supplementary file 1 [file Table_1.pdf]

Supplementary Table 1. Primers for miRNAs expression analysis

Primer name		Primers Sequences (5' to 3')
hsa-miR-125b-5p	RT primer	gtcgtatccagtgcagggtccgaggtattcgcactggatacgactcacaa
	Forward primer	tcgcatccctgagaccctaac
hsa-miR-182-5p	RT primer	gtcgtatccagtgcagggtccgaggtattcgcactggatacgacagtgtg
	Forward primer	tcgggtttggcaatggtagaact
hsa-miR-34a-5p	RT primer	gtcgtatccagtgcagggtccgaggtattcgcactggatacgacacaacc
	Forward primer	tcgcctggcagtgtcttagct
hsa-miR-143-3p	RT primer	gtcgtatccagtgcagggtccgaggtattcgcactggatacgacgagcta
	Forward primer	gcgaatgagatgaagcactg
hsa-miR-146a-5p	RT primer	gtcgtatccagtgcagggtccgaggtattcgcactggatacgacaaccca
	Forward primer	tcgcctgagaactgaattcca
hsa-miR-23b-3p	RT primer	gtcgtatccagtgcagggtccgaggtattcgcactggatacgacggtaat
	Forward primer	tcgccatcacattgccaggg
hsa-miR-301a-3p	RT primer	gtcgtatccagtgcagggtccgaggtattcgcactggatacgacgctttg
	Forward primer	tcgcccagtgcaatagtattgt
hsa-miR-21-5p/ mmu-miR-21-5p	RT primer	gtcgtatccagtgcagggtccgaggtattcgcactggatacgactcaaca
	Forward primer	tcgcctagcttatcagactga
hsa-miR-30c-5p	RT primer	gtcgtatccagtgcagggtccgaggtattcgcactggatacgacgctgag
	Forward primer	tcgcctgtaaacatcctacact
hsa-miR-29b-3p	RT primer	gtcgtatccagtgcagggtccgaggtattcgcactggatacgacaacact
	Forward primer	tcggctagcaccatttgaaatc
hsa-miR-16-5p	RT primer	gtcgtatccagtgcagggtccgaggtattcgcactggatacgaccgccaa
	Forward primer	cggactagcagcacgtaaata
hsa-miR-181b-5p	RT primer	gtcgtatccagtgcagggtccgaggtattcgcactggatacgacacccac
	Forward primer	tcgccaacattcattgctgtcg
hsa-miR-30b-5p	RT primer	gtcgtatccagtgcagggtccgaggtattcgcactggatacgacagctga
	Forward primer	tcgcctgtaaacatcctacac
hsa-miR-320a-3p	RT primer	gtcgtatccagtgcagggtccgaggtattcgcactggatacgactcgccc
	Forward primer	cggcaaaagctgggttgaga
Universal reverse primer		gtgcagggtccgaggt
U6	Forward primer	cgcttcggcagcacatatac
	Reverse primer	ttcacgaatttgcgtgtcat
